# Fibrinogen and base excess levels as predictive markers of the need for massive blood transfusion after blunt trauma

**DOI:** 10.1007/s00595-015-1263-7

**Published:** 2015-11-03

**Authors:** Takehiro Umemura, Yoshihiko Nakamura, Takeshi Nishida, Kota Hoshino, Hiroyasu Ishikura

**Affiliations:** Department of Emergency and Critical Care Medicine, Faculty of Medicine, Fukuoka University, 7-45-1 Nanakuma, Jonanku, Fukuoka, 814-0180 Japan

**Keywords:** Massive transfusion, Fibrinogen level, Scoring system, Blunt trauma patients

## Abstract

**Background:**

Assessment blood consumption and trauma-associated severe hemorrhage scores are useful for predicting the need for massive transfusion (MT) in severe trauma patients. However, fibrinogen (Fbg) and base excess (BE) levels might also be useful indicators for the need for MT. We evaluated the accuracy of prediction for MT of the scoring system vs. Fbg and BE.

**Methods:**

The subjects of this retrospective single center observational study were patients with injury severity score ≥16 trauma, divided into a non-MT group and an MT group. We compared variables, including the scoring system (comprising vital signs and focused assessment with sonography for trauma; FAST) and Fbg between the groups. We then performed a multiple logistic regression modeling and a receiver operating characteristic analysis to clarify which value was the most useful predictive indicator for MT.

**Results:**

There were 114 patients in the non-MT group and 39 in the MT group. The level of Fbg and BE were independent predictors of MT. The area under the curve values for Fbg and BE were 0.765 and 0.845, respectively, and the optimal cutoff values of Fbg and BE were 211 mg/dL and −1.4, respectively.

**Conclusions:**

Fbg and BE levels can be used as an independent predictor for MT.

## Introduction

Trauma is a leading cause of death in persons under the age of 40 and severe hemorrhage is a major cause of mortality in both civilian and military trauma [1–3]. Death from traumatic exsanguination usually occurs rapidly, typically in the first 6–12 h [4–6]. Approximately 10 % of these injured patients are transfused with one or more units of blood, and up to 30 % require a massive transfusion (MT), defined as 10 or more units of blood, in the first 24 h after admission [7, 8]. However, there are few simple criteria to predict the need for a massive transfusion (MT) in severe trauma patients. As previously reported, the assessment blood consumption (ABC) [9] and trauma-associated severe hemorrhage (TASH) [10] scoring systems are useful predictors of the need for MT. However, these scoring systems require the assessment of several factors, such as vital signs, focused assessment with sonography for trauma (FAST), pelvic fracture and/or femur fracture, making them slightly complicated. Therefore, we need to find some useful biomarkers for TM, which are easy to use in the emergency department.

Routine laboratory-based coagulation tests, such as prothrombin time/international normalized ratio (PT/INR), activated partial thromboplastin time (APTT) and fibrinogen (Fbg), platelet count, and hemoglobin (Hb) concentration are used to assess the patient’s coagulation status [11]. Schreiber et al. [12] reported that, in military casualties, coagulopathy was identified as a factor contributing to the need for MT. However, when severe bleeding occurs during surgery, coagulation factors do not decrease in a predictable manner. Conversely, it was recently reported that the Fbg level decreased earlier than other blood coagulation factors [13–15]. Stinger et al. [16] reported that the Fbg-to-Packed red blood cells (PRBCs) ratio (F/R) was independently associated with mortality [odds ratio (OR) 0.37, 95 % confidence interval (CI) 0.171–0.812, *p* = 0.013], and that the incidence of death from hemorrhage was significantly higher in the low-F/R ratio group than in the high-F/R ratio group, with mortality rates of 85 % (23/27) vs. 44 % (21/48), respectively, *p* < 0.001). They suggested that Fbg might play an important role in mortality and predict the need for MT. On the other hand, Kudo et al. reported that the Fresh frozen plasma (FFP)-to-PRBCs ratio during the first 6 h of admission might not affect mortality or morbidity [17]. Thus, there is not yet a unified view about whether Fbg administration affects the morbidity and mortality of severe trauma patients. Moreover, to our knowledge, there has been no investigation to define the optimal plasma Fbg level that would indicate if MT is required.

Based on the finding that base excess (BE) was a useful predictor of the need for volume replacement in trauma patients [18], this is now widely used in the evaluation of patients who have suffered severe trauma. We conducted the present study to evaluate the usefulness of other predictors of MT in comparison with previous scoring systems, such as the ABC and TASH scores, in patients with blunt trauma.

## Methods

This single center, retrospective study was conducted at Fukuoka University Hospital between June 2009 and December 2011. The study protocol was approved by the institutional review board of Fukuoka University Hospital according to the Declaration of Helsinki. Informed consent was waived in view of the retrospective and anonymous nature of the study. We enrolled blunt trauma patients with an injury severity score (ISS) ≥16 who were admitted to the hospital. MT was defined as the transfusion of 10 units or more of PRBCs in the first 24 h after admission. In Japan, one unit of PRBC represents a volume of about 120 mL. Patients who were expected to require more than 10 units of PRBC within 24 h, but did not survive for 24 h due to uncontrolled bleeding, were enrolled in the MT group to reduce survivor bias. The reason we enrolled these three patients in the MT group was based on the criteria of previous studies [19, 20] for the grouping of patients.

After allocating patients to an MT group or a non-MT group, we compared the two groups for age, sex, type of trauma (penetrating or blunt), ISS, Glasgow coma scale (GCS), systolic blood pressure (SBP) (mmHg), heart rate (HR) (/min), respiratory rate (RR) (/min), unstable pelvic fracture (UPF), femur fracture open/dislocated (FF O/D), FAST positive rate, hemoglobin (Hb) (g/dL), BE (mmol/L), ABC [9] and the TASH [10] scores, and Fbg level (mg/dL), INR, APTT (s), blood transfusion, operative management rate, and interventional radiology (IVR) at the time of admission. We also compared the 24 h and 28-day mortality rates between the groups and evaluated the usefulness of predictors for MT using logistic regression analysis.

### Blood samples

Blood samples were collected at the time of arrival at the ER and before the administration of fluids. Whole blood was collected with sodium citrate as an anticoagulant, in a conventional blood collection tube (NIPRO, Osaka, Japan). Several hemostatic markers, including the Fbg level, were measured within an hour after collection.

### Statistical analysis

Continuous variables are presented as the median and interquartile range (IQR). We used the Chi-square test (or Fisher’s exact test, whichever was appropriate) or the Wilcoxon test to compare the two groups. To identify the risk factors for MT, we performed univariate and multiple logistic regression modeling. A receiver operating characteristic (ROC) analysis was done to establish the most useful MT predictor. The Youden index was used to identify the cutoff values for levels of Fbg, BE, ABC score and TASH score, which may have MT significance. All analyses were calculated by the JMP^®^ version 10 (SAS institute, Tokyo, Japan). *p* values less than 0.05 were considered significant.

## Results

We enrolled 153 patients who had suffered severe blunt trauma within the given investigation period. There were 114 patients in the non-MT group and 39 patients in the MT group. Table 1 summarizes their demographic and clinical characteristics. Three patients who we included in the MT group were transfused with less than 10 units of PRBCs but died of uncontrolled massive bleeding within 24 h. The massive bleeding was from severe hemothorax in one, intra-abdominal hemorrhage secondary to liver injury in another one, and an unstable pelvic fracture in the other.

**Table 1:** Demographics and clinical characteristics of blunt trauma patients who did not need massive blood transfusion vs. those who did

Variables	Non-MT group (*n* = 114)	MT group (*n* = 39)	*p* value
Age, median (IQR)	58 (32 to 69)	57 (37 to 77)	NS
Male (%)	72.8	48.7	<0.01
Glasgow coma scale, median (IQR)	14 (12 to 15)	13 (7 to 14)	<0.05
SBP (mmHg), median (IQR)	130 (108 to 151)	109 (84 to 146)	<0.01
RR/min, median (IQR)	20 (18 to 24)	24 (17 to 28)	<0.05
HR/min, median (IQR)	84 (68 to 95)	103 (85 to 122)	<0.0001
ISS, median (IQR)	22 (17 to 25)	27 (22 to 34)	<0.01
FAST positive (%)	12.3	33.3	<0.01
Hb (mg/dL), median (IQR)	13.4 (11.6 to 15)	11.3 (9.2 to 12.9)	<0.0001
BE (mmol/L), median (IQR)	0.1 (−1.7 to 1.0)	−3.8 (−9.3 to −1.7)	<0.0001
Unstable pelvic fracture (%)	5.3	25.6	<0.001
Femur fracture open/dislocated (%)	4.4	12.8	NS
Penetrating (%)	1.8	2.6	NS
Antiplatelet argent (%)	7	0	NS
Anticoagulant argent (%)	3.5	2.6	NS
Liver disease (%)	0.9	0	NS
ABC score, median (IQR)	0 (0 to 0)	1 (0 to 2)	<0.0001
TASH score, median (IQR)	3 (1 to 4)	8 (5 to 13)	<0.0001
INR, median (IQR)	1.07 (1 to 1.17)	1.2 (1.05 to 1.43)	<0.001
APTT (s), median (IQR)	25.9 (23.9 to 28.7)	28.3 (25.8 to 37)	<0.01
Fbg (mg/dL), median (IQR)	241 (191 to 311)	167 (96 to 205)	<0.0001
PRBCs within 24 h U, median (IQR)	0 (0 to 3)	12 (10 to 20)	<0.0001
FFP within 24 h U, median (IQR)	0 (0 to 0)	10 (5 to 15)	<0.0001
PC within 24 h U, median (IQR)	0 (0 to 0)	10 (0 to 10)	<0.0001
Pelvic external rotation (%)	4.4	15.4	<0.05
Thoracotomy (%)	0.9	12.8	<0.01
Laparotomy (%)	2.6	7.7	NS
Craniotomy (%)	21.1	23.1	NS
IVR (%)	2.6	20.5	<0.001
24-h mortality (%)	5.3	38.5	<0.0001
28-day mortality (%)	6.1	46.2	<0.0001

*MT* massive transfusion, *IQR* interquartile range, *SBP* systolic blood pressure, *HR* heart rate, *ISS* injury severity score, *FAST* focused assessment with sonography for trauma, *Hb* hemoglobin, *BE* base excess, *ABC score* assessment of blood consumption score, *TASH score* trauma-associated severe hemorrhage score, *INR* international normalized ratio, *APTT* activated partial thromboplastin time, *Fbg* fibrinogen, *PRBCs* packed red blood cells, *FFP* fresh-frozen plasma, *PC* platelet concentrates, *IVR* interventional radiology

The RR, HR, ISS, FAST positive rate, UPF rate, ABC score, TASH score, INR, APTT, blood transfusion number (PRBCs, FFP, PC), pelvic external rotation rate, thoracotomy rate, laparotomy rate, IVR rate, 24 h mortality, and 28-day mortality were all significantly higher in the MT group. However, the ratio of male patients as well as SBP, Hb, BE, and Fbg levels were significantly lower in the MT group.

We evaluated the predictors of MT using a univariate logistic regression model, which revealed gender (male), HR (>120/min.), SBP (<90 mmHg), FAST positivity, UPF positivity, Hb, BE, and Fbg levels as useful predictors for MT (Table 2). We then performed multiple logistic regression modeling analysis. Explanatory values were selected by the significant predictors for MT in univariate analysis, which included gender, HR (<120/min vs. 120/min), SBP (<90 mmHg vs. 90 mmHg), FAST (positivity vs. negativity), UPF (positivity vs. negativity), Hb (g/dL), BE (mmol/L), and Fbg (mg/dL). From this result, a low level of Fbg and BE were independent predictors of MT (*p* < 0.01, *p* < 0.05, respectively; Table 3).

**Table 2:** Univariate logistic regression model for the predictors of massive transfusion

Variables of ABC and TASH scores	*p* value	OR	95 % CI
Sex (male vs. female)	<0.01	0.355	0.166–0.751
Mechanism (penetrating vs. blunt)	NS	1.474	0.067–15.799
Heart rate (≥120/min vs. <120/min)	<0.001	6.005	12.170–17.692
Systolic blood pressure (<90 vs. ≥90 mmHg)	<0.001	0.193	0.072–0.501
Focused assessment with sonography for trauma (positive vs. negative)	<0.01	3.571	1.490–8.589
Unstable pelvic fracture (positive vs. negative)	<0.001	6.207	2.129–19.606
Femur fracture open/dislocated (positive vs. negative)	NS	3.206	0.846–12.173
Hb (mg/dL)	<0.0001	0.716	0.605–0.834
BE (mmol/L)	<0.0001	0.767	0.681–0.847
Fbg (mg/dL)	<0.0001	0.987	0.981–0.992

*MT* massive transfusion, *OR* odds ratio, *CI* confidence interval, *Hb* hemoglobin, *Fbg* fibrinogen, *BE* base excess

**Table 3:** Multiple logistic regression model for the predictors of massive transfusion

Variables	*p* value	OR	95 % CI
BE (mmol/L)	<0.05	0.855	0.745–0.971
Fbg (mg/dL)	<0.01	0.992	0.986–0.998

Other variables entered in the model were: sex, systolic blood pressure (SBP) <90 vs. ≥90 mmHg, focused assessment with sonography for trauma (FAST) positive vs. negative, heart rate (HR) <120/min vs. ≥120/min, base excess (BE) (mmol/L), unstable pelvic fracture (UPF) positive vs. negative, hemoglobin (Hb) (mg/dL)

*MT* massive transfusion, *OR* odds ratio, *CI* confidence interval, *BE* base excess, *Fbg* fibrinogen

Finally, we performed the receiver operating characteristics (ROC) analysis, and calculated the area under the curve (AUC) to clarify which factor is the most useful predictor for MT. The highest AUC was achieved for BE (0.845) followed by the TASH score (0.833), Fbg (0.765), and then the ABC score (0.716). The optimal cutoff values for BE and Fbg were −1.4 mmol/L and 211 mg/dL, respectively. According to the AUC value, TASH and BE were significantly higher than the ABC score (*p* < 0.01, *p* < 0.01, respectively; Table 4), but not the Fbg level.

**Table 4:** Results of receiver operating characteristics analysis for massive transfusion

Variables	AUC	95 % CI	Optimal cutoff value	Sensitivity	Specificity	Positive predict value	Negative predict value
ABC score	0.724	0.628–0.803	1	64	78	25	89
TASH score	0.833*	0.744–0.895	5	77	77	30	88
Fbg (mg/dL)	0.765	0.683–0.898	211	78	65	34	81
BE (mmol/L)	0.845*	0.683–0.848	−1.4	80	67	31	76

*ROC* receiver operating characteristics, *MT* massive transfusion, *AUC* area under the curve, *CI* confidence interval, *ABC* assessment blood consumption, *TASH* trauma-associated severe hemorrhage, *Fbg* fibrinogen, *BE* base excess

* *p* < 0.01 vs. ABC score

## Discussion

Our study demonstrated that low levels of Fbg and BE were independent predictors of the need for MT in patients who have suffered severe blunt trauma. Both Fbg and BE levels are easy to measure and are already recognized as global markers. Consequently, there are already a few models being used to predict the need for MT, including the ABC score [9] and the TASH score [10]. However, these scoring systems include several variables, such as age, sex, vital signs, FAST, UPF, Hb, and BE, and require considerable time and effort to calculate the score. Hence, a simpler and faster indicator for MT in patients with active bleeding is needed.

This study showed that not only the BE level but also the Fbg level are independent predictive factors for MT. Recently, Yumoto et al. [20] reported that the BE level was a useful predictor of MT, while Soderlund et al. [21] found that mortality after blunt thoracic injury was closely associated with the BE level on admission. Some investigators also proposed that measures of hypoperfusion, such as lactate or BE levels, should be incorporated into the MT scoring systems [10, 22]. In fact, several studies about MT scoring systems have concluded that BE levels or serum lactate concentrations should be adopted as a component of the prediction of MT [10, 20, 22]. These reports strongly supported our results.

On the other hand, Fbg plays an essential role in the hemostatic coagulation systems linked to activated platelets and the formation of a fibrin network [23]. Additionally, a decrease in Fbg was more rapid than that in any other coagulation factor when plasma-poor blood transfusion or extracellular fluids were infused in patients with major blood loss [24]. When the Fbg level falls to 40 % of the normal level, a bleeding tendency ensues. On the other hand, a bleeding tendency does not develop when other coagulation factors (prothrombin, factor V, factor VII) fall to about 20 % of normal [24]. Inaba et al. [25] reported that the Fbg level increase correlates with improved survival rates for patients who required MT. Furthermore, they concluded that an Fbg level <100 mg/dL was a strong independent risk factor for death. It is well known that maintaining a high Fbg level improves the survival rate, but our study also suggests that the Fbg level is one of the most important predictors of the need for MT, in comparison with other coagulation factors, in trauma patients.

Our study demonstrated that the optimal cutoff value for the Fbg level on admission was 211 mg/dL (normal range 200–400 mg/dL with up to a 10-fold increase during tissue damage, infection, and cytokine-induced inflammatory responses [26], and with less than 100 mg/dL of Fbg predictive of microvascular bleeding [24]). Fenger-Eriksen et al. [26] reported that patients with serious bleeding, in whom a fibrinogen concentrate had been administered to treat hypofibrinogenaemia (defined by a laboratory threshold value below 200 mg/dL) had significantly less blood loss and transfusion requirements (RBC, FFP and platelets). This threshold is similar to that according to our results, which revealed that the level of Fbg for MT was less than 211 mg/dL in blunt trauma patients at the time of admission.

We included in our MT group, three patients who died of uncontrolled massive bleeding within 24 h. The Fbg levels were already extremely low in all three on arrival (89, 50, 163 mg/dL, respectively). This also suggested that a low Fbg level on admission might predict the clinical outcome of trauma patients.

In Japan, a portable Fbg analyzer CG02N (A&T Corporation, Kanagawa, Japan) has recently become available for clinical use. With this device, the Fbg level in a blood sample is available in about 1 min. We consider this mobile Fbg analyzer as a point of care testing in the emergency room and the bedside in the intensive care unit (ICU). We will attempt to validate the results of this study in relation to the usefulness of the Fbg level as predictor for MT by using CG02N in the near future.

This study has some limitations. First, it was retrospective and the sample size was small. Second, we did not consider the presence of severe head injury, although Lee et al. [27] reported recently that traumatic brain injury was not independently associated with more profound coagulopathy. Third, we could not examine the potential influence of the patient’s transport time and the fluids administered prior to admission to our center. Fourth, in this study we examined Fbg levels on admission only.

## Conclusions

This study demonstrated that Fbg and BE levels as a single factor could predict the need for MT. We recommend that Fbg and BE levels be measured on admission and as point of care testing, especially in patients who have suffered blunt trauma.
